# Neural State Monitoring in the Treatment of Epilepsy: Seizure Prediction—Conceptualization to First-In-Man Study

**DOI:** 10.3390/brainsci9070156

**Published:** 2019-07-01

**Authors:** Daniel John DiLorenzo, Kent W. Leyde, Dmitry Kaplan

**Affiliations:** 1 Department of Neurosurgery, Loma Linda University; Loma Linda, CA 92354, USA; 2 NeuroVista Corporation, Seattle, WA 98109, USA; 3 DiLorenzo Biomedical LLC, Seattle, WA 98109, USA; 4 Cascade Medical Devices, Sammamish, WA 98074, USA; 5 Kaplan Design LLC, Bellevue, WA 98008, USA

**Keywords:** intracranial monitoring, subdural electrodes, intracranial electrodes, seizure focus localization, chronic monitoring, epilepsy surgery, seizure monitoring, seizure prediction

## Abstract

This research study is part of a therapy development effort in which a novel approach was taken to develop an implantable electroencephalographic (EEG) based brain monitoring and seizure prediction system. Previous attempts to predict seizures by other groups had not been demonstrated to be statistically more successful than chance. The primary clinical findings from this group were published in a clinical paper; however much of the fundamental technology, including the strategy and techniques behind the development of the seizure advisory system have not been published. Development of this technology comprised several steps: a vast high quality database of EEG recordings was assembled, a structured approach to algorithm development was undertaken, an implantable 16-channel subdural neural monitoring and seizure advisory system was designed and built, preclinical studies were conducted in a canine model, and a First-In-Man study involving implantation of 15 patients followed for two years was conducted to evaluate the algorithm. The algorithm was successfully trained to correctly provide a) notification of a high likelihood of seizure in 11 of 14 patients, and b) notification of a low likelihood of seizure in 5 of 14 patients (NCT01043406). Continuous neural state monitoring shows promise for applications in seizure prediction and likelihood estimation, and insights for further research and development are drawn.

## 1. Introduction

Fear arising from the unpredictability of seizures, more so than the ictal event itself, presents a substantial impediment for patients with epilepsy [1]. To put this in perspective, for many patients, the time spent in ictus represents less than 1%; however, all of their remaining waking hours are impacted by the absence of awareness of when the ictus will occur. The presence of uncontrolled seizures significantly compromises the independence and employability of these patients, negatively impacting their quality of life more so than many other chronic conditions including hypertension, diabetes, and heart disease [2]. 

The existence of auras, the presence of non-aura symptoms noticed by the patients themselves [3] or the ability of patients to predict their seizures [4], subtle changes that may be detected by seizure alert dogs [5,6], and changes that may be noticed by family members hours or days prior to ictus [7] have provided rationale for the pursuit and identification of neural correlates of a pre-seizure state. A review by Dalziel and Uthman et al. found that in 31% of patients with dogs, the dogs were able to respond to the seizure and 10% could provide advance warning approximately three minutes in advance of the seizure [6], and Kirton et al. reported in a study of 22 patients with seizure response dogs (SRDs), that 59% (13 of 22) of the dogs exhibited seizure alerting behavior an average of 31 min in advance of ictal onset [8].

Over the past several decades, investigators have applied a variety of techniques in an attempt to predict seizures. Early approaches, such as that published in 1975 by Viglione et al., utilizing linear analyses of intracranial EEG (iEEG) with pattern recognition techniques to predict seizures was successful only in detecting seizures, but high false-positive rates limited its utility for seizure prediction [9]. 

In 1990, Iasemides, Sackellares, and colleagues demonstrated the use of a technique employing nonlinear dynamics or chaos theory to distinguish ictal and interictal phases and their transitions using data from a patient implanted with subdural electrodes [10]. Their subsequent work employing nonlinear dynamics to a larger database from five patients appeared to demonstrate seizure predictability [11], and further study led to publication in 2005 of the application of this to seizure prediction in data from two patients [12]. It was asserted that “the next seizure can be predicted 92.3% of the time, about 91 min prior to its onset, with the issue of 1 false warning every 8.27 h.”; however, these results have not been reproduced by others.

Due to the nature of the problem, that is the prediction of a recurring event with a specified prediction time horizon, a random process or “chance predictor” will be correct some of the time. Despite decades of research on the development of seizure prediction algorithms, a performance metric addressing overcoming this limitation had not been developed. No group had definitively demonstrated an algorithm that performed better than chance in the prediction of a seizure [13]. 

## 2. Translational Research and Development Effort

### 2.1. Epidemiology and Indications

Epilepsy is a heterogeneous group of potentially debilitating neurological disorders characterized by unpredictable recurring seizures. Estimates of the population of people with epilepsy worldwide are 69 million with a lifetime prevalence of epilepsy and 33 million with active epilepsy [14]. Among patients with epilepsy, approximately 70% have seizures controlled with medications alone, and the remaining 30% remain refractory to medical treatment, defined as control with one or two antiepileptic drugs. Focal onset seizures, and particularly seizures with an ictal onset in the temporal lobe, are often preceded by an aura. We hypothesized that temporal lobe epilepsy, being one of the most homogeneous of focal onset seizures and one which is commonly preceded with an aura, would be the most likely form of epilepsy to have a set of electrographic features with pre-ictal predictive value. Medically refractory temporal lobe epilepsy was therefore felt to be the indication for which the development of a predictive algorithm would most likely be successful. Since prior to this study, no prediction algorithm had been shown to provide statistically significant predictive value for any type of seizure; and since this was a pilot study, it was deemed prudent to preserve a relatively broad inclusion criteria for medically refractory partial seizures and/or secondarily generalized partial seizures. 

### 2.2. Development of an Appropriate Performance Metric

The absence of robust statistical grading criteria have limited the interpretation of many of these approaches. In 2008, as part of the Seizure Advisory System commercialization effort at NeuroVista, Snyder et al derived and published a proposed grading criteria for seizure prediction algorithms [13]. One of the limitations of previous seizure prediction studies and publications was the arbitrarily defined seizure prediction horizon. For a sufficiently long seizure prediction horizon and a sufficiently high seizure frequency, an arbitrary seizure prediction algorithm assessed with this method could be found to have a “seizure prediction” sensitivity that could approach 100%. To control for this potential limitation, Snyder et al applied a Poisson-process model to derive the expected performance of a chance predictor that generates variable duration warnings. They proposed a new test metric, the difference between algorithm sensitivity and chance sensitivity given an equal proportion of time spent under warning. Methods for hypothesis testing were also derived and verified by Monte Carlo analysis.

### 2.3. Database Assembly

In an effort to systematically develop a robust seizure prediction algorithm, the first step was the creation of a large database. Prior to this effort, published research done in academic settings was limited to algorithms developed on and tested with relatively small data sets, often with less than a dozen patients. Acquisition of quality data is expensive and time consuming, posing a constraint to some academic efforts with limited budgets. Similarly, a vast data set is essential to allow the development of robust algorithms that are applicable to the heterogeneous population of patients with various types of epilepsy. Furthermore, large data sets facilitate the essential partitioning of algorithm development into separate phases of training on one data set and testing on a distinct and separate data set; this is critical in order to avoid overfitting the algorithm. To this end, a data gathering and annotation clinical study was designed and carried out, and electrocorticographic (ECoG) data sets with video recordings were collected from over 200 patients undergoing intracranial subdural grid recordings at over a dozen private and academic medical centers throughout the US. As is done in the normal course of an invasive EEG monitoring session, complete video and EEG data sets were collected from inpatients, and Neurologists annotated the data sets, indicating the onset times of clinical seizures. Data sets were qualified such that they contained clean ictal and interictal data as well as verifiable clinical and electrographic seizures. Algorithms were developed and refined on a training dataset, and once developed, the algorithms were tested on a separate validation test library from 49 patient data sets with an average of four seizures per patient. 

### 2.4. Infrastructure Development

A distributed cluster computing system, named CASCADES (Cluster of Algorithm Simulation Computers and Digital EEG Storage) was designed and built by the NeuroVista engineering team in Seattle specifically for seizure prediction research. Storage requirements were based upon algorithm development using ECoG data from 50 to 100 patients, with estimated record lengths of 100 to 200 gigabytes per subject. Computational requirements were based upon the need to execute and evaluate hundreds of candidate algorithms, each comprising signal processing, feature extraction, classification, cross-validation, and performance metric calculation tasks. The final system includes 120 computing nodes with 675 gigaflops of processing power and 40 terabytes of on-line storage. Customized software was developed to manage algorithm development over this cluster system [15]. This was developed in 2005 when parallel cluster computing platforms were not generally available, and it represented, at the time, the largest computing environment dedicated to ictal event prediction research. This was an enabling resource, not available to other teams in academia or industry and custom developed at NeuroVista for the specific purpose of development of a seizure prediction algorithm.

### 2.5. Algorithm Development

The seizure advisory system (SAS) algorithm was designed by the NeuroVista engineering team to analyze finite intervals of periodically sampled ECoG from a multiplicity of electrodes. Each interval (or segment) of ECoG can be analyzed by a mathematical calculation (feature-extractor) that captures a particular aspect (feature) of the signal as a scalar value. Examples of simple feature values include the mean amplitude of ECoG from a single electrode, or the peak cross-correlation of ECoG from two different electrodes. From this definition, it is clear that the same feature-extractor can be applied to different electrode signals to calculate different features.

In excess of 5000 candidate feature-extractors were constructed and evaluated, comprising a plethora of ECoG signal biomarkers. To be useful, these features had to exhibit a change between a pre-seizure time window and an inter-ictal time window. The algorithm was being developed to simultaneously perform two functions: to predict the occurrence of seizures and to predict the lack of seizures. From this large set of candidate feature extractors, a much smaller set of 288 features were selected, representing a combination of 16 iEEG channels with unique filtering and analysis processes; these comprised the master set of feature-extractors for use in the SAS. Feature values were calculated by applying feature-extractors to the various electrode signals and assembling them into an n-element feature vector. The feature vector was analyzed by an n-dimensional classifier to provide a likelihood of seizure.

With a multiplicity of electrode signals and over 100 feature-extractors, the number of possible features is quite large. With 16 electrodes and 100 univariate feature extractors, for example, 1600 features are available. To avoid overfitting the high-dimensionality classifier to a limited dataset (“the curse of dimensionality”), the size of the feature vector was tightly constrained through a process of forward selection and backward elimination.

Combinations of vectors of feature-extractors were evaluated (through 10-fold block-wise cross-validation) to provide more robust seizure prediction, and the best of these combinations were tested on a different and second data set for verification. 

**Figure 1:**
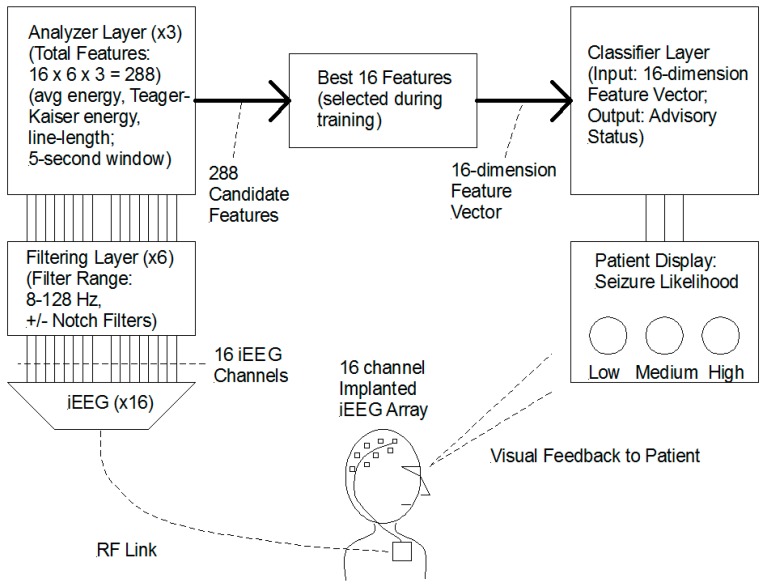
Block diagram depicting the functional structure of the Seizure Advisory System (SAS) Algorithm. A 16-channel implanted iEEG array provides real-time monitoring. A three-layer algorithm performs filtering, analysis, and classification. The combination of 16-channels (iEEG) with six filter options and three analyzer options, provides 288 possible features. During training, the 16 best performing features are selected for each patient. The classifier monitors the selected features and provides a real-time seizure estimate of seizure likelihood.

Once sufficient predictive functionality was achieved, the algorithm was implemented in an embedded microprocessor for the first-in-man (FIM) study. Each participant in the study received a personalized algorithm created using the feature selection and classifier training process described above. Figure 1 shows a simplified block diagram of the functional architecture of the Seizure Advisory System (SAS). 

The Seizure Advisory System (SAS) is a chronically implanted 16-channel continuous brain monitoring system with an external module that provides a visual indicator representing seizure likelihood to the patient. The implanted sensing electrodes comprise two electrode sets, each with two four-channel platinum-iridium subdural electrode strips encased in silicone. The electrodes are similar in geometry to subdural strip electrodes and may be positioned under the dura on the pial surface. The partitioning of the 16 electrodes into two pairs of four allows anatomically distant and distinct areas to be monitored; and the relative positioning of each pair of four is customizable as facilitated by the flexible cabling between each four-electrode strip. The electrodes may be positioned adjacent to a seizure focus and/or may be placed on the same or different lobes. This design facilitates substantial flexibility in electrode positioning and in signal selection. The surgeon may place all 16 electrodes in close proximity to the seizure focus, or alternatively, some electrodes may be placed distant from the focus and provide a source of relatively more normal signals for comparison. 

The implanted electronics include an implanted intracranial ECoG recording front-end and a real-time telemetering module. An external module worn by the patient contains a processor in which the SAS algorithm was implemented and which provided notification to the patient of a high, intermediate, or low seizure likelihood, through lighting a red, white, or blue indicator. 

**Figure 2:**
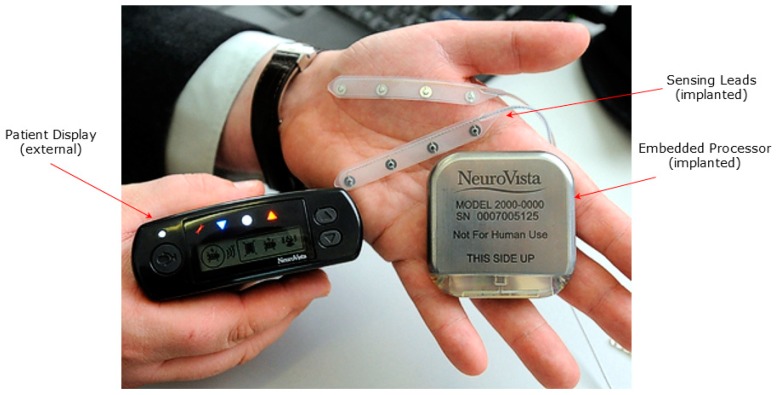
Seizure Advisory System (SAS) shown with implanted subdural electrodes, implanted EEG processor and telemetry unit, and external patent advisory module with red, white and blue indicator lights.

For algorithm training facilitated by the Data Collection Phase, the external hand-held device (Patient Module) performed data recording for subsequent customized algorithm design. This data was then sent to NeuroVista, where the data was annotated with clinical events and then run through the master algorithm on a distributed cluster computing network. The algorithm was trained through forward selection and backward elimination to optimize the selection of features for each patient. The trained algorithm was then downloaded onto the subject’s hand-held device (Patient Module). During the Advisory Stage, the Patient Module ran the SAS algorithm and notified the patient of the seizure likelihood. For each subject, the Patient Module was fully capable of calculating all of the features from the master algorithm; however, only a small and patient-specific subset were enabled for each subject. This system is shown in Figure 2.

This SAS technology was first tested in a canine model with naturally occurring epilepsy [16]. The primary goal was the assessment of the feasibility of continuous ambulatory intracranial EEG (iEEG) monitoring. Secondary goals included (1) the recording of ictal events in dogs and to assess their similarity to human seizures on iEEG and (2) to test system components and the surgical procedure and its postoperative tolerability. Six dogs were implanted, and a total of 11,671 h of iEEG data was collected from 31 July 2009 to 31 December 2009. Infrequent interruptions in the transmitted RF signal rendered roughly 3.8% of the data uninterpretable, leaving 96.2% of the data, or 11,232 h, available for analysis. Two of these dogs had video EEG (vEEG) monitoring performed to capture ictal events. Electrode impedances remained in the 4 to 15 kilohm range, facilitating low recording noise (1–2 microvolts RMS). Both ictal and interictal activity recorded from canines was found to be similar to that in humans. A seizure detection algorithm performed comparably on the canine data as on the human data base derived from Epilepsy Monitoring Unit (EMU) recordings [16].

This SAS technology was incorporated into a clinical grade implant, and human testing was initiated in a First-In-Man (FIM) study at three centers in Australia in which 15 patients were implanted with the Seizure Advisory system (SAS) [17]. This study was registered with ClinicalTrials.gov and was assigned Identifier: NCT01043406. Patients were recruited from three centers in Victoria, Australia: Royal Melbourne Hospital (Melbourne, Victoria, Australia, 3050), St. Vincent’s Hospital (Melbourne, Victoria, Australia, 3065), and Austin Health (Melbourne, Victoria, Australia, 3081). 

The study began in March 2010 and concluded in October 2012, and the inclusion and exclusion criteria are shown below:

#### Inclusion Criteria:

1. The subject has disabling partial seizures and/or secondarily generalized partial seizures. Disabling refers to seizures that are severe enough to cause injuries or to significantly impair areas of function such as employment, psychological or social wellbeing, or mobility.

2. The subject has failed treatment with a minimum of two AED’s used in typical therapeutic dosages.

3. For three months prior to enrollment, the subject’s anti-epileptic medication dosages have been stable, and the subject has had at least two disabling seizures per month, on average, with a seizure-free interval not to exceed 45 days. Seizures must be separated by a minimum of eight hours not to be considered part of a cluster. A cluster, for the purpose of this criterion, shall be considered a single seizure.

#### Exclusion Criteria:

1. For three months prior to enrollment, the subject’s anti-epileptic medication dosages have not been stable, or subject has had more than 12 disabling seizures per month, on average, or there was a seizure-free interval longer than 45 days. Clinical seizures must be separated by a minimum of eight hours to not be considered part of a cluster. A cluster, for the purpose of this criterion, shall be considered a single seizure.

2. The subject is implanted with pacemaker, implantable cardiac defibrillator, cardiac management product, or a medical device that interferes with the SAS or with which the SAS interferes. This includes, but is not limited to, direct brain neurostimulators, spinal cord stimulators, vagus nerve stimulators (VNS), and cochlear implants. Patients with a vagus nerve stimulator implanted but turned off through the duration of the study may be enrolled, provided their clinical status has been stable for at least one month with VNS turned off.

3. The subject has been diagnosed with primary generalized seizures.

Among the 15 patients with partial seizures with or without secondary generalization, the epileptogenic zones were temporal in four patients, frontotemporal in five, parietotemporal in four, and occipitoparietal in two. The age range was 20 to 61 (mean 44.5), and the gender composition was 60% male and 40% female. Prior surgical resection had been performed in 40% (6 of 15) of the patients, of which three had been temporal and three had been frontotemporal [17]. 

**Figure 3:**
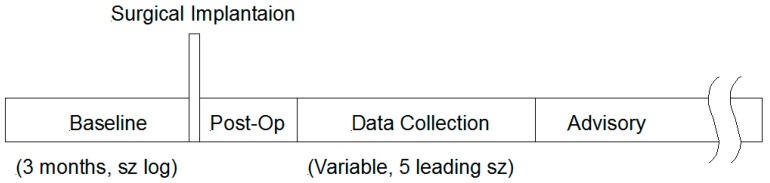
Study Timeline: After enrollment, the patients commence a three month baseline phase involving diligent seizure log maintenance, followed by surgical implantation of the SAS, post-op recovery, matriculation into a Data Collection Phase, then qualified entry into an Advisory Phase. Algorithms were tested both during the Data Collection Phase and the subsequent Advisory Phase.

Each patient underwent a three month baseline period, as shown in the Study Design Timeline in Figure 3, during which seizure activity was logged by the patient. This baseline period was part of the screening process, and only patients meeting the inclusion criteria (i.e., 2–12 seizures per month on average, no seizure free interval ≥ 45 days, seizures or seizure clusters separated by ≥ 8 h) were enrolled in the study and subsequently implanted. 

**Figure 4:**
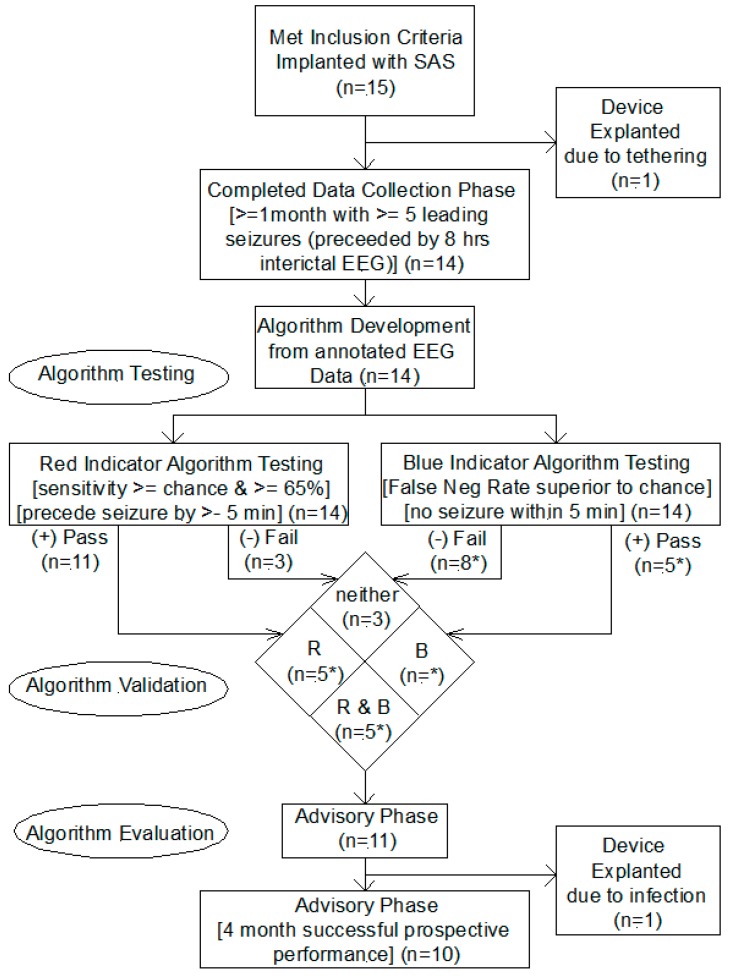
Study Design Flow Diagram: This represents the flow of the 15 patients who were implanted with an SAS through the Data Collection Phase, then through an algorithm testing and validation step, followed by algorithm evaluation in an Advisory Phase.

Patients were then implanted with the SAS, as shown in the Study Design Flow Diagram in Figure 4; and after a postoperative recovery period, the patients entered a Data Collection Phase of variable length and which for completion required the presence of at least five leading seizures in one month, leading seizures comprising a clinically correlated seizure with at least eight hours of preceding interictal EEG recording. The telemetered intracranial EEG data was annotated with seizure events using patient and device logs, including patient activated and automatic device activated audio recordings which allowed for the verification of clinical seizure events using patient and witness voice recordings, producing clinically correlated seizures. Of the 15 enrolled patients, 14 completed this phase and one required explantation due to tethering of the subcutaneous wire. 

Using clinically correlated seizures from the Data Collection Phase, the algorithm was trained for each patient, producing a customized algorithm with three outputs, (1) red indicator (high likelihood of seizure), (2) white indicator (intermediate likelihood of seizure), and (3) blue indicator (low likelihood of seizure). If either the red or the blue algorithm met performance criteria, the patient advanced to the Advisory Phase for evaluation of SAS efficacy. The performance criteria for the red algorithm was sensitivity greater than chance and greater than 65% and for the blue algorithms was False Negative Rate superior to chance [17]. Fourteen of the fifteen patients implanted completed the data collection phase and advanced to the algorithm validation phase, for 11 of which the red or blue indicator algorithms met criteria and advanced to the Advisory Phase. The breakdown of these subjects who advanced is shown in the “Algorithm Validation” section of Study Design Flow Diagram in Figure 4. The algorithm performance for one patient (Patient 4 in Cook 2013 [17]) was not available since that patient was discontinued due to explantation for infection. The uncertainty is indicated for the numbers marked with an asterisk. The pass & fail rates for the blue indicator are shown as 5* & 8*, respectively; this could be 6 & 8 or 5 & 9, depending on the status for Patient 4.) 

All 11 patients completing the Algorithm Validation phase began the Advisory Phase, and eight of these 11 completed the Advisory Phase Efficacy assessment at the four month follow up, revealing two patients in whom all seizures occurred in the red high likelihood state, demonstrating 100% sensitivity for these patients. 

## 3. Results

### 3.1. Summary of Patient Details and Algorithms Validation and Testing

Table 1 shows a summary of the 15 patients enrolled into the study and implanted. Source data is from the clinical paper [17]. The demographics, location of seizure focus, AEDs, and prior surgical resection or implantation of VNS are shown. The pre-implantation patient reported seizure rate and the iEEG-determined seizure rates are shown and compared. A very poor correlation between these numbers is shown, casting doubt on the reliability of patient seizure diary logs. To quantify this discrepancy, the ratio of the Seizure Frequency at Enrollment as determined by the patients log (SF_Pt) to the Seizure Frequency as captured on EEG (SF_EEG) was calculated, and this discrepancy ratio (Pt/EEG) ranges from 0.06 to 13.51, varying by over two orders of magnitude. 

As shown in Figure 4 and Table 1, one of the 15 patients implanted with the SAS had the device removed due to tethering and resulting discomfort. The remaining 14 patients completed the Data Collection Phase and underwent algorithm training. Of these 14 patients, the algorithms for 11 passed the validation criteria; and thee failed to achieve sufficient performance by the “high likelihood” algorithm and were explanted. One of the first tasks accomplished in this R & D effort was the development of a new and valid metric for characterizing the performance of a prediction algorithm which compared the algorithm performance in the context of seizure frequency and prediction horizon to chance. This was published by Snyder et al in 2008 [13] and was used in the validation of algorithm performance.

Of the 11 patients who entered the advisory phase, explantation was required in one due to damage to a lead during a surgical evacuation of an infection, leaving 10 patients to reach the four month endpoint in the Advisory Phase. By design, the “High Likelihood” algorithm met performance criteria in all 10 of these patients, and the “Low Likelihood” algorithm met criteria in five of these 10 or 50% of those completing the advisory phase. 

In two patients, all of the seizures occurred in the “High Likelihood” time, garnering a 100% sensitivity. Interestingly, these patients are also the two with the lowest frequency of seizures, each having been estimated as three seizures per month prior to implantation and each in which three seizures were predicted in the advisory stage.

The performance of the “Low likelihood” algorithm, in the 50% of the patients in whom it was validated, was remarkable, providing a positive predictive value (PPV) of 100% in four patients and 98% in one patient.

To characterize a representation of the specificity, the percentage of time during which the SAS algorithm indicated a “high risk’ and “low risk” was calculated, and these are shown in the table Similarly, a “Likelihood Ratio”, defined as Likelihood ratio = ([number of events in high advisory]/[time in high advisory])/([number of events in moderate advisory]/[time in moderate advisory]) was calculated and is also shown in Table 1 [17]. 

### 3.2. Seizure Prediction and Likelihood Seizure Advisory Efficacy

**Figure 5:**
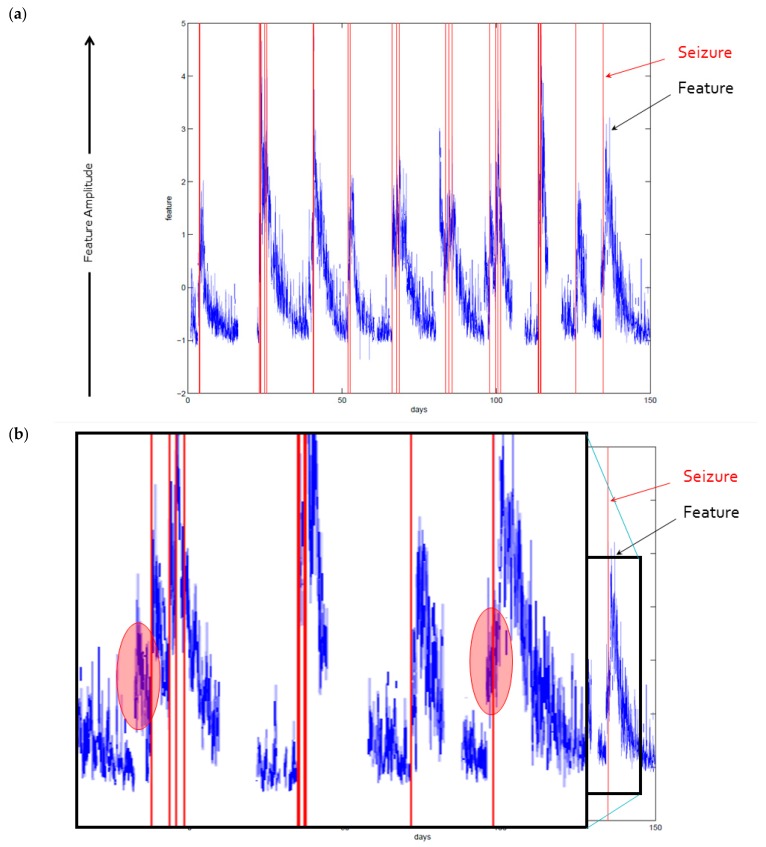
(**a**): Example feature shown over 150-day span. The feature is shown as a continuous value in blue, and seizure vents are marked as discrete vertical lines in red. Though in this feature, post-ictal changes are apparent for extended durations following each seizure, shorter interval yet detectable and actionable changes are also seen preceding the seizures. (**b**): A magnified portion of the feature plot is shown as an inset. In the two regions highlighted with a partially transparent red oval, changes in the feature are evident prior to the onset of the seizure.

As found in the algorithm development stage, features from the implanted SAS iEEG recordings were found to have predictive value. Figure 5 shows one of the more recently developed and promising features over a 150 day span, in which the feature magnitude changes prior to the onset of each seizure. 

**Figure 6:**
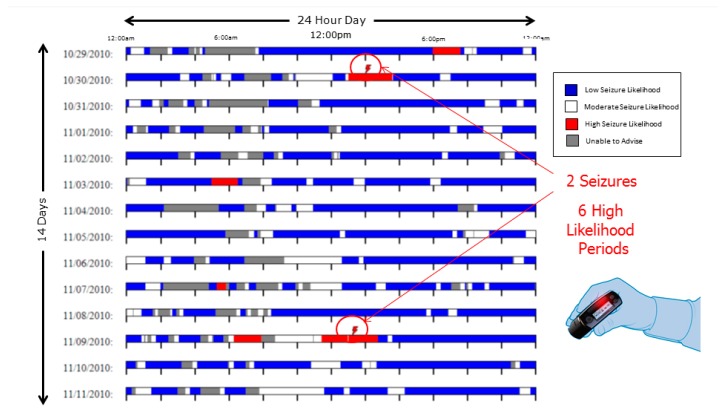
Advisory Timeline over 14 day contiguous block. Each of the 14 horizontal rows represents a continuous 24 h period. Each day-long row is a raster plot comprising columns of pixels, each column representing 2.3 min duration and comprising a vertical series of pixels, each pixel representing 13.8 s. In this time Advisory Timeline block, the patient had two seizures, each of which occurred within a “High Likelihood” (red indicator) advisory, of which there were six in this 14 day period.

Seizure prediction sensitivity was found to be 100% in two patients at four months follow up. In Figure 6, one data set spanning 14 contiguous days demonstrates 100% sensitivity. 

**Figure 7:**
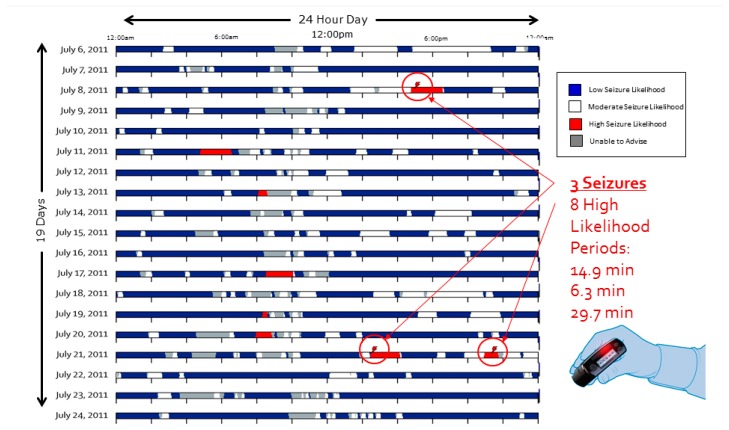
Advisory Timeline over 19 day contiguous block. Each of the 19 horizontal rows represents a continuous 24 h period. Each day-long row is a raster plot comprising columns of pixels, each column representing 2.3 min duration and comprising a vertical series of pixels, each pixel representing 13.8 s. In the Advisory Timeline block, the patient had three seizures, each of which occurred within a “High Likelihood” (red indicator) advisory, of which there were eight in this 19-day period. In chronological order, the seizure advisory advance warning times were 14.9, 6.3, and 29.7 min.

In Figure 7, a separate data set extending across 19 contiguous days also demonstrating 100% sensitivity. 

Of the five patients in whom the blue indicator was enabled, four achieved 100% negative predictive value and the fifth achieved 98%. The mean advance seizure warning time provided to the 10 subjects who advanced to the Advisory Stage was 114 min (range 5–960) [17].

### 3.3. Seizure Logs

The EEG and audio monitoring features of the SAS enable accurate determination of the occurrence and timing of seizures. Substantial discrepancies were noted between the monthly seizure frequencies as logged by the patients and as determined using SAS data [17]. The difference between the patient logged and the clinically confirmed electrographic seizures ranged by more than an order of magnitude in both directions (ratio of logged to CCS was 0.06 to 13.51).

### 3.4. Nocturnal Seizures and Medication Dosing

In the study, one patient who had reported being drowsy and having impaired cognitive function in the morning was found to be having large numbers of unreported nocturnal seizures. Use of such a system may facilitate customized antiepileptic drug (AED) dosing, providing a means for further optimizing the efficacy versus side effect tradeoff often inherent in AED regimens.

### 3.5. Interaction of Medications with Features

For a subset of features, there appeared to be sensitivity to antiepileptic drug (AED) levels, and some features appeared to lose sensitivity with increasing AED levels. To maintain sensitive and stable prediction algorithm function, features that tended to show variability with AED levels were excluded from those in the final patient-specific algorithm.

### 3.6. Drift of Algorithm and Feature Performance

Many of the patients required periodic retraining of the algorithm, on the order of every four months. Overall algorithm performance tended to remain stable with the benefit of retraining. This suggests but does not prove that the overall patient-specific algorithm and/or perhaps the features themselves should adapt to disease progression. Longer term studies will be required to ascertain this.

### 3.7. Importance of Quality Annotated Data

From the outset, it became very clear that one of the rate limiting steps in the development of a quality and high performance algorithm was the availability of vast quantities of high quality annotated data. While this need is shared by much of supervised learning algorithms, the particular requirements of this project (i.e. the desire to predict both the likelihood of an ictal event occurring and it not occurring) make the algorithm development process particularly sensitive to both false positive and false negative labels in the dataset. A non-labeled event is as damaging to the performance as an incorrectly labeled non-event. Generating this data requires substantial effort and resources since fully annotated data, in which every seizure is labeled, is not routinely produced in the clinical workflow.

### 3.8. Importance of Long-Term Monitoring and Recordings

Data generated from present EMU sessions typically consists of approximately one week duration recordings. Usually, only a small number of seizures are captured within this short time frame, leaving a small number of seizures to be used for training and testing. A data base of long-term recordings, such as those generated in the FIM study with this device and other long term recording systems, would allow development and testing of algorithms on the same patient, without the need to pool seizure recordings together and test mean performance, a constraint imposed by EMU short-duration recording sessions. Further, medication taper in the EMU setting introduces non-stationarity, a situation overcome in a long-term ambulatory monitoring setting.

### 3.9. Resective Epilepsy Surgery Planning

In one patient in the SAS FIM study, in whom the algorithm did not meet criteria and the patient did not progress to the Advisory Phase, sufficient data was recorded from the Data Collection Phase to allow the identification and localization of a previously unidentified focal cortical dysplasia. This finding guided subsequent successful resective surgery. This finding is consistent with that shown by DiLorenzo et al. in 2014 in a series of four patients implanted with a Responsive Neurostimulation (RNS) with uncontrolled seizures who were monitored chronically and subsequently underwent successful resective surgery guided by the intracranial EEG recordings [18,19]. This represents a new and beneficial application of chronic monitoring, nominally intended for other purposes, for detailed seizure characterization and potentially surgical planning in patients who may otherwise not be considered candidates for surgery. 

## 4. Discussion

The utility of chronic intracranial neural monitoring is demonstrated for several applications, some of which were expected or intended and others of which were novel or serendipitous findings. 

In the novel FIM study involving the NeuroVista SAS device, the algorithm was found to be highly sensitive in some patients, reaching 100% sensitivity in two of the 11 that made it to the advisory phase. The mean seizure advance warning time of 114 min is substantial, more than sufficient for the patient to take an intramuscular, sublingual, or even an oral antiepileptic drug in order to potentially prevent the occurrence of the predicted seizure. 

Even higher degrees of efficacy were realized with the blue indicator (low seizure likelihood) with four of five having 100% and one having a 98% negative predictive value. Each of these functionalities may be life changing for the patient, enabling them to engage in relatively risky activities (walking outside, perhaps swimming or sports, or other activity) with the confidence that they will not be caught off guard by a seizure. 

Predictive information or even likelihood estimates may be used in dosing medications for the patient. This may open the doors to entirely new ways of dosing medications for patients with epilepsy and which achieve higher seizure control rates with lower risks of side effects including sedation and impairment of cognitive performance.

The use of chronic recording for seizure planning, as shown in both the SAS study as well as in the RNS study, demonstrates the value of continuous chronic recording outside of the hospital environment and in an ambulatory setting.

With the initial demonstration of the real-world clinical efficacy shown by this system in a chronic long-term ambulatory setting, efforts have intensified to develop seizure prediction algorithms and systems. To this end, databases similar to those used by NeuroVista have been assembled and made publicly available for research teams to test algorithms. Epilepsy centers in Bonn, Germany (http://epileptologie-bonn.de/cms/front_content.php?idcat=193) and in Freiberg, Germany (http://epilepsy.uni-freiburg.de/freiburg-seizure-prediction-project/eeg-database) as well as the Children’s Hospital in Boston (http://www.physionet.org/pn6/chbmit/) have provided EEG databases for public use [20]. In 2008, the EPILEPSIAE project received funding from the European Union to develop a large EEG database. This project included six partner hospitals, universities, and companies in Germany, France, Italy, and Portugal and assembled a database including data sets from 275 patients [20], comparable in size to that built by NeuroVista; and it is being used in the development of pilot ambulatory seizure prediction systems [21].

This study was a First in Man clinical study and as such was relatively small (15 patients) and of modest duration (three patients with two year follow up). The clinical paper and this more technical follow up paper likely raise more questions than they answer. Additional, much larger studies with longer duration follow up are required to better characterize the performance of prediction algorithms and their efficacy in the multiplicity of seizure subtypes in the heterogeneous population of patients that are diagnosed with epilepsy. In contrast to many other physiological and neurological monitoring fields, the sheer number of degrees of freedom to this problem (epilepsy diagnoses, previous surgical treatments, previous and current medical treatments, anatomical variability, seizure frequency and regularity, epileptiform activity propagation speed and patterns, etc.), to the device (number and geometrical configuration of electrodes, amplifier bandwidths, sampling rates, etc.), and algorithm solutions (numbers and types of features, single and differential signal inputs to features, algorithm training processes, algorithm dynamic adaptability, etc.), and to the statistical analyses, makes this a rich area of inquiry deserving of extensive further study.

## 5. Conclusions

Seizure prediction and prediction of seizure likelihood appear possible with existing technology. Further development and evaluation of chronic neural recording technologies may fundamentally advance new treatment modalities as well as provide direct improvement in quality of life for its patients.

## 6. Patents

9,788,744Michael Bland Kent W. Leyde, Neil G. McIlvaine, Shan Gaw, Peter Weiss, John F. Harris, Systems for monitoring brain activity and patient advisory device, Filed July 28, 2008, Issued October 17, 20179,643,019Jason A. Higgins, Michael Bland, Kent W. Leyde, W. Douglas Sheffield, John F. Harris, David M. Himes, Neurological monitoring and alerts, Filed February 14, 2011, Issued May 9, 20179,622,675Kent W. Leyde, John F. Harris, Communication error alerting in an epilepsy monitoring system, Filed March 23, 2011, Issued April 18, 20179,592,004Daniel J. DiLorenzo, Kent W. Leyde, Methods and systems for managing epilepsy and other neurological disorders, Filed March 28, 2016, Issued March 14, 20179,480,845John F. Harris, Kent W. Leyde, Nerve stimulation device with a wearable loop antenna, Filed March 10, 2015, Issued November 1, 20169,445,730David Snyder; Kent W. Leyde, John F. Harris, Implantable systems and methods for identifying a contra-ictal condition in a subject, Filed September 9, 2013, Issued September 20, 20169,421,373Daniel John DiLorenzo, Apparatus and method for closed-loop intracranial stimulation for optimal control of neurological disease, Filed November 15, 2006, Issued August 23, 20169,415,222Daniel John DiLorenzo, Monitoring an epilepsy disease state with a supervisory module, Filed July 21, 2008, Issued August 16, 20169,375,573Daniel John DiLorenzo, “Systems and methods for monitoring a patient’s neurological disease state”, Filed September 23, 2005, Issued June 28, 20169,320,900Daniel John DiLorenzo, “Methods and systems for determining subject-specific parameters for a neuromodulation therapy”, Filed December 29, 2006, Issued April 26, 20169,289,595Jared Floyd; Christopher Genau; Kent W. Leyde, Medical lead termination sleeve for implantable medical devices, Filed November 18, 2013, Issued March 22, 20169,259,591David Brown, Christopher Genau, Kent W. Leyde, Shan Gaw, Jeffrey Stewart, Housing for an implantable medical device, December 23, 2008, Issued February 16, 20169,113,801Daniel John DiLorenzo, “Methods and systems for continuous EEG monitoring”, Filed December 29, 2006, Issued August 25, 20159,044,188Daniel John DiLorenzo, “Methods and systems for managing epilepsy and other neurological disorders”, Filed May 12, 2014, Issued June 2, 20159,042,988Daniel John DiLorenzo, “Closed-loop vagus nerve stimulation”, Filed November 17, 2005, Issued May 26, 20158,868,172Kent W. Leyde, Daniel John DiLorenzo, “Methods and systems for recommending an appropriate action to a patient for managing epilepsy and other neurological disorders”, Filed December 28, 2005, Issued October 21, 20148,855,775Kent W. Leyde, Systems and methods of reducing artifact in neurological stimulation systems, Filed October 23, 2012, Issued October 7, 20148,849,390Javier Ramon Echauz, David E. Snyder, Kent E. Leyde, Processing for multi-channel signals, December 29, 2009, Issued September 30, 20148,786,624Javier Ramon Echauz, David E. Snyder, Kent E. Leyde, Processing for multi-channel signals, Filed 8,781,597Daniel John DiLorenzo, “Systems for monitoring a patient’s neurological disease state”, Filed May 5, 2010, Issued July 15, 20148,762,065Daniel John DiLorenzo, “Closed-loop feedback-driven neuromodulation”, Filed June 22, 2005, Issued June 24, 20148,725,243Daniel John DiLorenzo, “Methods and systems for recommending an appropriate pharmacological treatment to a patient for managing epilepsy and other neurological disorders”, Filed December 28, 2005, Issued May 13, 20148,588,933Jared Floyd; Christopher Genau; Kent W. Leyde, Medical lead termination sleeve for implantable medical devices, Filed January 11, 2010, Issued November 19, 20138,543,199David Snyder; Kent W. Leyde, John F. Harris, Implantable systems and methods for identifying a contra-ictal condition in a subject, Filed September 2, 2011, Issued September 24, 2013 8,396,557Daniel John DiLorenzo, “Extracranial monitoring of brain activity”, Filed Apr 4, 2012, Issued Mar 12, 20138,295,934Kent W. Leyde, Systems and methods of reducing artifact in neurological stimulation systems, Filed November 14, 2006, Issued October 23, 20128,036,736David Snyder; Kent W. Leyde, John F. Harris, Implantable systems and methods for identifying a contra-ictal condition in a subject, Filed March 21, 2008, Issued October 11, 20117,930,035Daniel John DiLorenzo, “Providing output indicative of subject’s disease state”, Filed May 2, 2007, Issued April 19, 20117,853,329Daniel John DiLorenzo, “Monitoring efficacy of neural modulation therapy”, Filed December 29, 2006, Issued December 14, 2010D627,476Shan Gaw, Michael Bland, Peter D. Weiss, Kent W Leyde, John F. Harris, Medical patient advisory device, Filed August 29, 2007, Issued November 16, 20107,747,325Daniel John DiLorenzo, “Systems and methods for monitoring a patient’s neurological disease state”, Filed September 28, 2005, Issued June 29, 20107,676,263John F. Harris, Kent W. Leyde, Jaideep Mavoori, Minimally invasive system for selecting patient-specific therapy parameters, Filed June 21, 2007, Issued March 9, 20107,623,928Daniel John DiLorenzo, “Controlling a subject’s susceptibility to a seizure”, Filed May 2, 2007, Issued November 24, 20097,403,820Daniel J., DiLorenzo, “Closed-loop feedback-driven neuromodulation”, Filed May 25, 2005, Issued July 22, 20087,324,851Daniel J., DiLorenzo, “Closed-loop feedback-driven neuromodulation”, Filed June 1, 2004, Issued January 29, 20087,277,758Daniel J., DiLorenzo, Methods and systems for predicting future symptomatology in a patient suffering from a neurological or psychiatric disorder, Filed April 5, 2004, Issued October 2, 20077,242,984Daniel J., DiLorenzo, Apparatus and method for closed-loop intracranial stimulation for optimal control of neurological disease, Filed January 6, 2004, Issued July 10, 20077,231,254Daniel J., DiLorenzo, Closed-loop feedback-driven neuromodulation, Filed July 12, 2004, Issued June 12, 20077,209,787Daniel J., DiLorenzo, Apparatus and method for closed-loop intracranial stimulation for optimal control of neurological disease, Filed November 20, 2003, Issued April 24, 20076,819,956Daniel J., DiLorenzo, Optimal method and apparatus for neural modulation for the treatment of neurological disease, particularly movement disorders, Filed November 11, 2001, Issued November 16, 20046,366,813Daniel J., DiLorenzo, Apparatus and method for closed-loop intracranial stimulation for optimal control of neurological disease, Filed June 25, 1999, Issued April 2, 2002

## Figures and Tables

**Table 1:** Patient Demographics, Algorithm Development Phases, Algorithm Performance. See text for description. Data from clinical paper [17]. (KEY: AEDs: CAR = Carbamazepine, CLO = Clonazepam, LAC = Lacosamide, LEV = Levetiracetam, LAM = Lamotrigine, OXC = Oxcarbazepine, PER = Perampanel, PHE = Phenytoin, RET = Retigabine, TOP = Topiramate, VAL = Valproate, ZON = Zonisamide. Notes include: IFXN: Patient discontinued the study prior to completing the four month initial follow up in the Advisory Phase due to explantation related to infection. The infection was evacuated, but incidental damage to the lead required removal. REM: Device removal due to adverse event (migration with tethering of subcutaneous wire causing discomfort, prompting patient to request explantation) prevented completion of data collection phase. FAIL: Algorithm failed to meet Red Indicator (high likelihood) performance criteria required to progress to Advisory Phase, and the device was explanted. VNS: For the single patient with a VNS system, the VNS was explanted when the SAS was implanted. Likelihood ratio = ([number of events in high advisory]/[time in high advisory])/([number of events in moderate advisory]/[time in moderate advisory]). ALL: All events occurred during the high likelihood advisory (infinite ratio). (PLAN): Device failed to meet criteria for algorithm performance, but chronic monitoring facilitated subsequent identification & resection of a seizure focus. (PSYCH): Some seizures were found to be psychogenic. (NOCT): Monitoring with SAS revealed nocturnal seizures (10–20/night), felt to be the cause of daytime drowsiness.)

Patient Number and Demographics							Seizure Rate			Algorithm Development Phases						Seizures		Algorithm Performance							Note or Side Effects
Patient #	Age	Gender	Epileptogenic Zone	Prior Treatment			Estimate at Enrollment (SF_Pt)	Captured by iEEG (SF_EEG)	Discrepancy: Ratio Pt/EEG	Data Collection		Validation		Advisory		Clinically Correlated Seizures (CCS)	Both Clin Equivalent & Corr Sz (CES+CCS)	High Likelihood Sensitivity		Low Likelihood Neg Predictive Value (%)	Time in Advisory		Likelihood		
				Prior surgery						Failure Reason	Completed (1 = y,0 = n)	Failure Reason	Completed (1 = y,0 = n)	Failure Reason	Completed (1 = y,0 = n)						High (%)	Low (%)	Ratio (CCS)	(CES + CES)	
				Resection	VNS	Antiepileptic Drugs (AEDs)												(CCS) (%)	(CES + CCS) (%)						
1	26	M	PT			CLO, LEV, LAM, VAL	4	14.17	0.28		1		1		1	7	13	86%	77%	100%	27%	7%	14.3	8.0	
2	44	M	OT			LAC, LAM, OXC, VAL	3	1.52	1.97		1		1		1	3	3	100%	100%	100%	31%	56%	ALL	ALL	
3	22	F	PT	1		CAR, LAM, PHE	7	126.65	0.06		1		1		1	58	106	56%	45%		29%	N/E	3.1	2.1	(NOCT)
4	61	M	PT			CAR, LAC, LAM, TOP, PHE	5	3.61	1.39		1		1	IFXN	0										IFXN (Infection, drainage w damage, removal)
5	20	F	FT	1		CLO, LAM, OXC, TOP	4	1.32	3.03	REM	0		0		0										Migrated (removal)
6	62	M	T			none	2	6.32	0.32		1	FAIL	0		0										
7	52	M	FT			CAR, CLO, LEV					1	FAIL	0		0										(PLAN)
8	48	M	FT	1	1	CAR, LEV	4	42.32	0.09		1		1		1	36	86	63%	62%		28%	N/E	4.4	4.2	
9	51	F	OP			CAR,	10	30.37	0.33		1		1		1	49	52	18%	17%	98%	11%	48%	0.8	0.8	
10	50	F	FT	1		LEV, OXC, ZON	4	52.28	0.08		1		1		1	109	164	54%	51%		17%	N/E	5.8	5.1	
11	53	F	FT			LAC, PHE, PER	8	102.50	0.08		1		1		1	11	39	56%	39%	100%	15%	26%	5.1	2.6	
12	43	M	T			LAM, LAC, PHE, RET	5	0.37	13.51		1	FAIL	0		0										(PSYCH)
13	50	M	T	1		CAR, CLO, LEV, LAC	7	25.74	0.27		1		1		1	26	113	57%	50%		28%	N/E	3.4	5.1	Seroma (drainage)
14	49	F	PT			CLO, OXC	3	0.00			1		1		1	3	3	100%	100%	100%	3%	88%	ALL	ALL	
15	36	M	T	1		CAR, LAC, PER, TOP	5	6.28	0.80		1		1		1	21	24	71%	71%		41%	N/E	3.6	3.5	
Avg:	44.5		Number:	6			5.07	29.53	0.06	Number:	14	Number:	11	Number:	10	32.3	60.3	66%	61%	99.6%	23%	45%	5.1	3.9	
			%:	40%			Avg	Avg	13.51	%:	93%	%:	73%	%:	67%										
